# Particle-Size-Dependent Delivery of Antitumoral miRNA Using Targeted Mesoporous Silica Nanoparticles

**DOI:** 10.3390/pharmaceutics12060505

**Published:** 2020-06-02

**Authors:** Lisa Haddick, Wei Zhang, Sören Reinhard, Karin Möller, Hanna Engelke, Ernst Wagner, Thomas Bein

**Affiliations:** 1 Department of Chemistry and Center for NanoScience, University of Munich (LMU), Butenandtstrasse 5-13, 81377 Munich, Germany; lisa.haddick@cup.lmu.de (L.H.); K.Moeller@lmu.de (K.M.); hanna.engelke@cup.lmu.de (H.E.); 2 Pharmaceutical Biotechnology and Center for NanoScience, University of Munich (LMU), Butenandtstrasse 5-13, 81377 Munich, Germany; wei.zhang@cup.uni-muenchen.de (W.Z.); reinhard.soeren@cup.uni-muenchen.de (S.R.); Ernst.Wagner@cup.uni-muenchen.de (E.W.)

**Keywords:** mesoporous silica nanoparticles, core-shell, surface functionalization, cell targeting, size-dependent delivery, antitumoral microRNA (miRNA), confocal microscopy

## Abstract

Multifunctional core-shell mesoporous silica nanoparticles (MSN) were tailored in size ranging from 60 to 160 nm as delivery agents for antitumoral microRNA (miRNA). The positively charged particle core with a pore diameter of about 5 nm and a stellate pore morphology allowed for an internal, protective adsorption of the fragile miRNA cargo. A negatively charged particle surface enabled the association of a deliberately designed block copolymer with the MSN shell by charge-matching, simultaneously acting as a capping as well as endosomal release agent. Furthermore, the copolymer was functionalized with the peptide ligand GE11 targeting the epidermal growth factor receptor, EGFR. These multifunctional nanoparticles showed an enhanced uptake into EGFR-overexpressing T24 bladder cancer cells through receptor-mediated cellular internalization. A luciferase gene knock-down of up to 65% and additional antitumoral effects such as a decreased cell migration as well as changes in cell cycle were observed. We demonstrate that nanoparticles with a diameter of 160 nm show the fastest cellular internalization after a very short incubation time of 45 min and produce the highest level of gene knock-down.

## 1. Introduction

A novel modality in cancer therapy emerged over the past two decades in the form of gene therapy and in this context, small interfering RNA (siRNA) and micro RNA (miRNA) are promising alternatives to common anti-cancer medications [1,2]. Both nucleotide oligomers mediate the process of RNA interference (RNAi) in which cells use these short RNA strands of 19-24 nucleotides to recognize messenger RNA (mRNA) with a complementary sequence, induce their destruction and thus inhibit the translation into proteins. To provide cancer therapy, synthetically produced siRNA and miRNA mimics can be used that target and silence specific oncogenes. Recent studies have identified a number of cancer-related genes as potential targets for RNAi-based therapy [3,4,5]. One of these tumor suppressor miRNA is miR200c, which targets the proto-oncogene KRAS. It regulates cell differentiation, proliferation and survival [6], the epithelial to mesenchymal transition [7] and suppresses chemoresistance [8].

However, a major challenge for a widespread therapeutic application of RNAi is creating appropriate delivery vehicles to safely and effectively deliver and release siRNA and miRNA into the cytosol of disease-causing cells. Several excellent reviews summarize the requirements for siRNA delivery and comprehensively report on the efficiencies of gene silencing achieved with various delivery materials [9,10,11].

Some promising results were recently obtained from ongoing clinical trials concerning RNA delivery vehicles and in 2018 Alnylam’s Onpattro (Patisiran) has been FDA-approved as the first ever RNAi drug against nerve damage caused by the rare hereditary disease, transthyretin amyloidosis [12]. However, Patisiran is a lipid-based nanocarrier system that does not actively target the cancer tissue, and most of the administered drug accumulates passively in the liver instead. Therefore, robust and specifically targeted carrier systems are needed for an efficient delivery of siRNA and miRNA to prevent a premature degradation of the unstable RNA. For this purpose, nanoparticles have attracted much attention because they provide a stable, nontoxic, and highly flexible platform.

Mesoporous silica nanoparticles (MSN) are emerging as potential nanocarriers for a variety of anti-cancer cargos including nucleic acids [13,14]. The properties that have rendered them specifically suitable for siRNA and miRNA delivery include a high surface area and pore volume, a controllable biodegradability [15,16], the tunability of particle size and pore size, their variable morphology and importantly, the possibility to create core-shell particles constructed of spatially separated regions with different surface functionalization [17,18].

This tunability of MSNs opens the possibility to systematically study the influence of the carrier properties on the gene silencing efficacy. Multiple parameters can affect successful gene silencing using carrier agents, e.g., the adsorption and release kinetics of the nucleic acid in the drug carrier, the dependence of cellular uptake or endosomal escape on nanoparticle size, surface characteristics such as charge and/or the attachments of functional residues, to name a few, but systematic studies addressing these issues are rare [19].

For the adsorption and release kinetics of nucleic acids using MSNs, the pore size, pore morphology and surface charge are important parameters. High loadings of nucleic acid were achieved by using large pore MSNs with a pore diameter of around 10–20 nm [20] and when pores were modified with cationic functional groups—aminopropyltriethoxysilane (APTES) or polylysine—to create a cationic layer for nucleic acid adsorption [14,21]. However, some of these systems suffer from poor release kinetics associated with low knockdown efficacies because of either the high affinity of polycations towards nucleic acid or because of a bottleneck-type pore morphology that features smaller pore openings than pore diameters, which both might decrease an efficient release of the highly charged nucleic acid molecules. Based on these findings, our group introduced novel medium-pore MSNs (pore diameter of around 5 nm) with stellate pore morphology, which were able to adsorb an exceptionally high amount of siRNA of 380 µg mg^−1^ and which enabled a mainly electrostatically driven fast and efficient RNA desorption, resulting in a high silencing efficacy [21].

Cellular internalization of the nanocarrier is another factor that influences gene silencing efficacy. The relationship between shape and size of MSNs [22,23,24,25] or other nanocarriers [26,27] and cellular internalization is important as nanoparticle size may affect the uptake efficiency and kinetics and the internalization mechanism [28]. One of the great advantages of MSNs is that they can be designed to feature different sizes and shapes. For MSNs [22,29] as well as for Au [30], polystyrene [31] and iron oxide nanoparticles [32], a size-dependent uptake in cells was observed with a maximum uptake at a particle diameter of 30–50 nm. However, as size is only one among several parameters controlling cellular uptake of nanoparticles, optimal sizes may vary for different surfaces and different surface functionalizations. Additionally, targeting ligands were shown to improve selective cellular uptake and a better accumulation in tumor tissue [33,34]. The effect of particle size on gene transfection efficiency using silica-based nanoparticles with diameters from 125 to 570 nm as nanocarriers for plasmid DNA was studied by Yu et al. Here, the transfection efficiency was found to be a compromise between binding capacity of the nanocarriers and cellular uptake. Smaller particles showed higher cellular uptake but less binding capacity for plasmid DNA. Particles with a diameter of 330 nm showed the best gene transfection efficacy [35].

Gene silencing mediated by delivery of siRNA and miRNA with MSN nanocarriers is strongly dependent on the escape of siRNA or miRNA from the endosomes into the cytosol. To trigger this reaction, the surface of particles is often decorated with cationic polymers, which are known to support an endosomal escape. Polyethylenimine (PEI) is one well-studied polymer exhibiting good endosomal escape capability when used at high dosage, attributed to a ‘proton sponge’ effect, however, it has a poor toxicity profile [36]. For instance, the group of Gu showed very good knockdown efficacies when PEI was attached to the outside of their particles [37,38].

A systematic study of the correlation between gene knockdown efficacy and endosomal escape kinetics was performed by Wang et al. [39]. They used magnetic MSNs capped with PEI at low concentrations to avoid toxic effects, resulting in poor endosomal escape ability. When endosomal escape was induced by a chloroquine treatment at early transfection times they could still see a high gene silencing efficacy. In contrast, when applied at later stages, a low gene-silencing efficacy was found, presumably because the released RNA was already degraded within the endosome.

**Figure 1:**
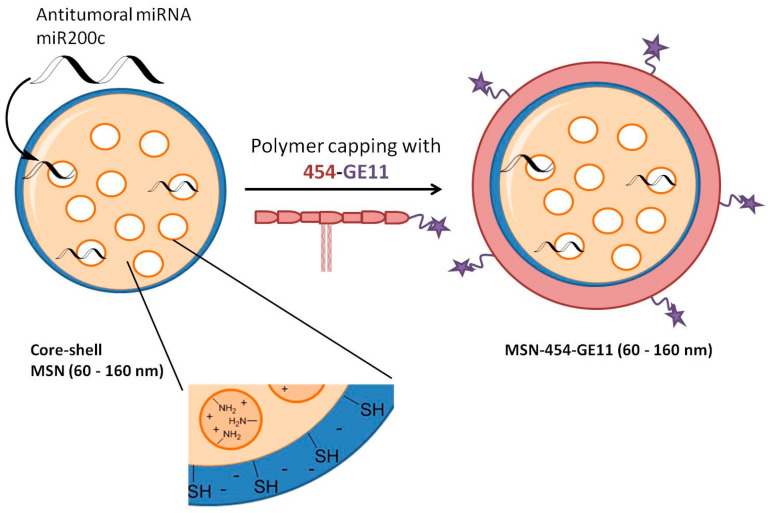
Schematic overview of the structure of core-shell mesoporous silica nanoparticles (MSN), the loading of miR200c and the polymer capping resulting in the MSN-454-GE11 vector. The positively charged core in core-shell MSN enables a high loading capacity for miRNA. The mercapto-lined MSN shell associates with a positively charged block copolymer carrying the targeting ligand GE11, thus acting simultaneously as capping, endosomal release and targeting agent.

Although MSNs have shown great potential as an efficient carrier system for siRNA and miRNA delivery, systematic studies about particle properties and corresponding gene-silencing efficacy remain rare. Herein, we present a systematic investigation of the particle size-dependent delivery of miRNA using core-shell MSNs for gene silencing in T24 cells. A series of MSNs with uniform sizes ranging from 60 to 160 nm with an average pore diameter of around 5–6 nm was used to encapsulate an antitumoral microRNA mimic (miR200c) or a control RNA with a scrambled sequence (Ctrl). The core of these particles was functionalized with APTES to yield a positively charged inner surface to accommodate the negatively charged miRNA, while a thinner surface layer was enriched with mercaptosilane, forming a negatively charged shell to enable binding of the capping agent. This consisted of an amino-acid block-copolymer 454 (see Figure 1) to aid endosomal escape and which was further linked via polyethylene glycol (PEG) to the peptide GE11, targeting the epidermal growth factor receptor [40]. This vector is abbreviated as MSN-454-GE11. An alternative construct for the delivery of this miRNA was studied before by Müller et al. using RNA-encapsulating polyplexes consisting of the same copolymer 454 functionalized with GE11 [41]. Their particles, featuring a size of 120–150 nm, successfully showed antitumoral effects with two different therapeutic RNAs. 

Our series of MSN-454-GE11 particles covers a broad size range but still provides comparable properties for each sample, including surface chemistry, surface charge, pore size and miRNA concentration to enable a conclusive study of the particle-size effect on miRNA delivery. Here, we show that good gene-silencing and antitumoral effects are obtained when miR200c is delivered by the largest MSN-454-GE11 particles in this sequence. 

## 2. Materials and Methods

### 2.1. Reagents and Materials

2-(*N*-morpholino)ethanesulfonic acid (MES, Sigma-Adrich, Darmstadt, Germany), 3-aminopropyltriethoxysilane (APTES, Sigma-Adrich), 3-mercaptopropyl triethoxysilane (MPTES, >95%, Sigma-Adrich), ammonium fluoride (NH_4_F, >98% Fluka, Darmstadt, Germany), Atto 633-carboxy dye (Atto-Tec, Siegen, Germany), 4′,6-diamidino-2-phenylindole (DAPI, Thermo Fisher, Schwerte, Germany), block copolymer surfactant (Pluronic F127 (EO_106_PO_70_EO_106_), Sigma-Aldrich), cetyltrimethyl-ammonium chloride (CTAC, 25% in H_2_O, Sigma-Adrich), *N*-(3-dimethylaminopropyl)-*N*′-ethylcarbodiimide hydrochloride (EDC, >97%, Sigma-Adrich), *N*-hydroxysulfosuccinimide (sulfo-NHS, 98%, Sigma-Adrich), octadecyltrimethylammonium bromide (C_18_Br, Sigma-Adrich), propidium iodide, tetraethylorthosilicate (TEOS, >98%, Sigma-Aldrich), triethanolamine (TEA, 98%, Fluka), triisopropylbenzene (TiPB, 96%, Sigma-Adrich). Oligomer 454 [42] and Mal-PEG-GE11 [41] were synthesized as described before. siRNA and miRNA duplexes were purchased from Axolabs GmbH (Kulmbach, Germany): control RNA (sense: 5-AuGuAuuGGccuGuAuuAGdTsdT-3′, antisense: 5′-CuAAuAcAGGCcAAuAcAUdTsdT-3), miR200c (sense: 5′-UCCAUCAUUACCCGGCAGUAUUA-3, antisense: 5′-UAAUACUGCCGGGUAAUGAUGGA-3). 

### 2.2. Synthesis of MSN-NH2in-Shout

Two slightly different synthesis methods were used to prepare core-shell functionalized MSN containing 4.5% amino groups in the core and 2% thiol groups on the shell (MSN-NH_2in_-SH_out_).

Procedure A: Core-shell MSN particles were prepared by a co-condensation reaction according to our previous reports [17,43]. Early reports from our group showed that a consistent decrease in particle size can be obtained by decreasing the amount of TEA serving as organic base. This concept was applied for the synthesis of samples MSN160 nm, MSN130 nm and MSN100 nm [44]. A mixture of TEA (14 g, 93.8 mmol/10 g, 67 mmol/4 g, 26.8 mmol, respectively), tetraethylorthosilicate (TEOS, 1.74 mL, 7.84 mmol) and 3-aminopropyltriethoxysilane (APTES, 89 µL, 0.38 mmol) in a polypropylene reactor was heated in an oil bath at 90 °C for 20 min without stirring. A second solution was prepared consisting of water (2.41 mL, 1.21 mol), CTAC (2.71 mL, 1.83 mmol), TiPB (2.97 mL, 12 mmol) and ammonium fluoride (NH_4_F, 100 mg, 2.7 mmol) and was heated to 60 °C under stirring. The second solution was subsequently added to the first solution under strong stirring. The whole mixture was further stirred at room temperature for 20 min. After this time, four portions of TEOS (4 × 51 µL, 0.92 mmol) were added in three minute intervals and the solution was stirred for another 30 min. To prepare the shell layer, a premixed solution of TEOS (42 µL, 0.190 mmol) and mercaptopropyl triethoxysilane (MPTES) (42 µL, 0.22 mmol) was added to the whole mixture. The condensation reaction was allowed to continue over night at room temperature. 

Procedure B: MSN80 nm and MSN60 nm were prepared using the tri-block copolymer F127 as a particle growth inhibitor/dispersant. The procedure is based on an adapted recipe reported in the literature [45]. First, a mixture of TEA (7 g, 46.8 mmol/4 g, 26.7 mmol, respectively), TiPB (1.5 mL, 6.2 mmol), F127 (100 mg, 8 µmol) TEOS (2 mL, 9 mmol), and APTES (89 µL, 0.38 mmol) was prepared in a polypropylene reactor and heated at 90 °C for 20 min. Separately, NH_4_F (100 mg, 2.7 mmol) and C_18_Br (0.35 g, 0.9 mmol) were dissolved in H_2_O. This solution was heated to 90 °C under static conditions for 30 min and was then added under strong stirring to the first solution at once. The combined solutions were further heated at 60 °C for 30 min. The ingredients for the shell layer, TEOS (42 µL, 0.190 mmol) and MPTES (42 µL, 0.22 mmol), were mixed, preheated to 60 °C and added to the reaction mixture. Heating at 60 °C was continued for 30 min, and then the reaction mixture was allowed to cool down to room temperature under stirring and was continuously stirred overnight. 

### 2.3. Template Extraction

Agglomerates were removed from the as- synthesized samples by centrifugation for a short time at low speed (7197 rcf, 5 min). The pellet was discarded and the remaining particles in the supernatant were collected via centrifugation (7197 rcf, 20 min) for template extraction. After centrifugation, the particles were resuspended in a solution containing 2 g ammonium nitrate in 100 mL ethanol and heated for 45 min under reflux (90 °C), cooled and collected by centrifugation (7197 rcf, 20 min). The first reflux treatment was followed by a second extraction step for 45 min under reflux (90 °C) using a solution of 10 mL concentrated hydrochloric acid in 90 mL ethanol. The MSNs were collected by centrifugation for 20 min (7197 rcf) and washed with ethanol (3 × 70 mL). Finally, MSNs were dispersed in 15 mL ethanol and used for further characterization. 

### 2.4. Characterization

For transmission electron microscopy (TEM), samples were prepared by drying a diluted ethanolic suspension of MSN on a carbon-coated copper grid at room temperature for several hours. The measurements were performed on a Tecnai G2 20 S-Twin instrument operated at 200 kV with a TVIPS TemCam-F216 camera. 

Dynamic light scattering (DLS) and zeta potential measurements were performed with a Malvern Zetasizer Nano instrument equipped with a 4 mW He-Ne-Laser (633 nm). For DLS data, a diluted colloidal suspension of the particles was measured in PMMA cuvettes at 25 °C. For zeta potential measurements, the additional Zetasizer titration system (MPT-2) was used based on diluted NaOH and HCl as titrants. For this purpose, a colloidal suspension of MSNs in water at a concentration of 0.1 mg mL^−1^ was prepared. 

Raman spectroscopy was performed on a Bruker Equinox 55 with FRA-106 Raman attachment with a ND:YAG laser (1064 nm) and a laser power of 100 mW. Infrared spectra of dried sample powder were recorded on a ThermoScientific Nicolet iN10 IRmicroscope in reflection–absorption mode with a liquid-N_2_ cooled MCT-A detector. 

A Quantachrome Instrument NOVA 4000e was used for nitrogen sorption analysis at −196 °C. Sample outgassing was performed for 12 h at 120 °C and at a vacuum of 13.3 × 10^−3^ mbar. A quenched solid density functional theory (QSDFT) equilibrium model of N_2_ on silica at a relative pressure *p/p*_0_ = 0.8 was used to calculate the pore size and pore volume, based on the adsorption and desorption branch of the isotherm. A BET model in the range *p/p*_0_ = 0.05–0.2 was used to determine the specific surface area. 

Thermogravimetric analysis (TGA) of the powder samples (10 mg) was performed on a Netzsch STA 440 C TG/DSC using a heating rate of 10 °C/min up to 900 °C with a stream of synthetic air of about 25 mL·min^−1^. siRNA concentrations were determined by UV measurements performed with the Nanodrop 2000c spectrometer (Thermo Scientific).

### 2.5. Loading of Ctrl and miR200c for Gene Silencing

In all experiments, 100 µL Ctrl or miR200c solution (c = 50 ng µL^−1^) in MES buffer (pH = 5) was added to 100 µg MSN-NH_2in_-SH_out_ samples, resulting in a final RNA concentration of 50 µg mg^−1^ of MSN carrier. Samples were vortexed and shaken at 37 °C for 30 min to complete adsorption. Particles were washed via centrifugation and were resuspended in 100 µL HEPES buffer. The supernatant was collected to verify complete adsorption of RNA. 

### 2.6. Loading of Fluorescent Dye for Cellular Internalization Studies

A total of 1 mL MSN-NH_2in_-SH_out_ samples (pH = 5; 1 mg mL^−1^ MES buffer) was mixed with 2 µL Atto-633 carboxy (2 mg mL^−1^ in anhydrous DMSO), 10 µL EDC and a catalytic amount of sulfo-NHS. The samples were vortexed and shaken for 4 h, were washed multiple times afterwards with MES and HEPES buffer (1 mL, respectively) (centrifugation steps: 10 min, 16,900 rcf) and were redispersed in 1 mL MES buffer.

### 2.7. Capping with 454-GE11

For capping of MSN-NH_2in_-SH_out_ samples, 10 µL Mal-PEG-GE11 reagent (5 mg mL^−1^ in H_2_O) was added to 100 µL 454 solution (5 mg mL^−1^ in H_2_O) and shaken for 2 h at 37 °C. Then, 454-GE11 was mixed with 1 mL MSN suspension (1 mg mL^−1^ in HEPES buffer) and again shaken for 2 h at 37 °C. Resulting MSN-454-GE11 samples were centrifuged (16,900 rcf, 10 min) and washed with water and HEPES buffer. The samples were redispersed in 2 mL HEPES buffer.

### 2.8. miRNA Binding Assay

For the miRNA binding assay, a 2.5% agarose gel was prepared by dissolving agarose in TBE buffer (Trizma base 10.8 g, boric acid 5.5 g, disodium EDTA 0.75 g, and 1 L of water) and by heating to 100 °C. GelRed^®^ (1:10,000) was added and the agarose gel was cast in the electrophoresis unit. MSN-454-GE11 samples loaded with Ctrl RNA were mixed with 4 µL loading buffer (6 mL glycerol, 1.2 mL of 0.5 M EDTA, 2.8 mL H_2_O, 0.02 g bromophenol blue) and placed into the gel sample pockets. Electrophoresis was run at 120 V for 90 min.

### 2.9. Cell Culture

HeLa, T24 or T24/eGFPLuc-200cT (authenticated by DSMZ, Braunschweig, Germany) cells, stably expressing an eGFP-luciferase fusion gene under the control of the CMV promoter and with a target site for miR200c, were cultivated in Dulbecco’s modified Eagle’s medium (DMEM) supplemented with 10% fetal bovine serum (FBS), 100 U mL^−1^ penicillin and 100 U mL^−1^ streptomycin. 

### 2.10. Cell Viability Determined by MTT Assay

MTT assay was performed in triplicate in 96-well plates. One day prior to transfection, T24 cells were seeded at 3500 cells/well. Before transfection, medium was replaced by 80 µL fresh medium. MSN samples (20 µL) loaded with or without RNA were added at different concentrations in HEPES buffer and incubated for 45 min at 37 °C followed by a medium exchange. Subsequently, cells were returned to the incubator and 48 h after transfection a viability assay was performed. For this, an MTT solution (100 µL/well; 0.5 mg mL^−1^ in DMEM) was added for 2 h. Then, the supernatant was removed and cells were lysed by freezing at −80 °C. DMSO (100 µL) was added and plates were incubated at 37 °C under shaking. Absorption at 590 nm against a reference wavelength of 630 nm was measured using a SpectraFluorTM Plus microplate reader S4 (Tecan, Groeding, Austria). Cell viability was calculated as percentage of absorption compared to wells treated with HEPES buffer. 

### 2.11. Cellular Adhesion Determined by Flow Cytometry

T24/eGFPLuc-200cT cells were seeded 24 h before transfection on 24-well plates with a density of 5 × 10^5^ cells per well in 1000 µL growth medium. After 24 h, the medium was replaced by 400 µL fresh medium. A total of 100 µL MSN, MSN-454-PEG and MSN-454-GE11 samples loaded with fluorescent dye (500 µg mL^−1^ MSN in HEPES buffer) were added and incubated for 45 min at 37 °C. After incubation time, the cells were washed three times with PBS, 500 I.U. heparin to remove particles non-specifically associated to the cell surface and were detached with trypsin/EDTA, taken up in growth medium, centrifuged and resuspended in PBS containing 10% FBS. Cellular internalization of the MSN samples was assayed by flow cytometry at an Atto-633 carboxy fluorescent dye excitation wavelength of 635 nm and detection of emission at 665 nm. Cells were gated by forward/sideward scatter and pulse width for exclusion of doublets. DAPI (4′,6-diamidino-2-phenylindole) was used to discriminate between viable and dead cells. Data were recorded by BD LSRFortessa™ (BD Biosciences, USA) and analyzed by FlowJo^®^ 7.6.5 flow cytometric analysis software. All experiments were performed in triplicate.

### 2.12. Confocal Fluorescence Microscopy

Fluorescence microscopy was performed with a Zeiss Observer SD spinning disk confocal microscope using a Yokogawa CSU-X1 spinning disc unit and an oil objective (63× magnification) and BP 525/50 (WGA488) and LP 690/50 (Atto633) emission filters. A 488 nm and a 639 nm laser were used for excitation. At 24 h prior to transfection, 3500 T24 cells per well were seeded in 8-well plates in 280 µL growth medium. A total of 20 µL MSN-454-GE11 covalently labeled with Atto-633 carboxy (200 µg mL^−1^ MSN in HEPES buffer) were added to each well and incubated for 45 min or 6 h, respectively, at 37 °C followed by addition of WGA-488 to the medium for cell membrane staining. Cells were washed with PBS and after addition of 300 µL fresh medium, cells were directly imaged. 

The cellular uptake of nanoparticles was quantified using the ImageJ macro “Particle_in_Cell-3D” developed by Adriano A. Torrano and Julia Blechinger, Department of Chemistry and Center for NanoScience (CeNS, University of Munich (LMU), Munich, Germany. http://imagejdocu.tudor.lu/doku.php?id=macro:particle_in_cell-3d)

### 2.13. Luciferase Gene Silencing

In all experiments, siRNA and miRNA delivery was performed in 96-well plates in triplicate. 3500 T24/eGFPLuc-200cT cells per well were seeded 24 h prior to transfection in 100 µL growth medium. Before transfection, medium was replaced with 80 μL fresh growth medium. 20 µL MSN, MSN-454-PEG and MSN-454-GE11 for RNA delivery (500 µg mL^−1^ MSN in HEPES buffer) were added to each well and incubated for 45 min at 37 °C followed by a medium exchange. Cells were then incubated with 100 µL fresh medium for an additional 48 h following transfection. After this time, cells were treated with 100 μL cell lysis buffer (Promega (Mannheim, Germany)). Luciferase activity in cell lysate (35 μL) was measured using a luciferin-LAR (1 M glycylglycine, 100 mM MgCl_2_, 500 mM EDTA, DTT, ATP, coenzyme A) buffer solution on a luminometer for 10 s (Centro LB 960 plate reader luminometer, Berthold Technologies, Bad Wildbad, Germany).

### 2.14. Cell Cycle Analysis

At 24 h prior to transfection, 3.5 × 10^4^ T24 cells per well were seeded in a 12-well plate in 1000 µL growth medium and then medium was replaced by 400 µL fresh medium. A total of 100 µL MSN-160 nm-454-GE11 (500 µg mL^−1^ in HEPES buffer) loaded with Ctrl RNA or miR200c was added and incubated at 37 °C for 4 h. After a selected incubation time, the medium was replaced with fresh medium and cells were incubated for an additional 72 h. Afterwards, cells were washed with PBS and detached with trypsin/EDTA. Cells were washed with PBS twice and suspended in 100 µL PBS. The cell suspension was added dropwise to 0.9 mL cold 70% ethanol and incubated at 4 °C for 2 h. Then, cell pellets were suspended in 1 mL PBS after centrifugation and incubated for 15 min at room temperature for counting. A total of 1 × 10^5^ cells of each sample was incubated in 300 µL propidium iodide/TritonX-100 containing RNase solution for 15 min at 37 °C. For cell cycle analysis, the cells were analyzed by flow cytometry at an excitation wavelength of 488 nm and detection of emission with a 613/20 bandpass filter. Data were recorded by BD LSRFortessa™ (BD Biosciences, USA) and analyzed by FlowJo^®^ 7.6.5 flow cytometric analysis software. All experiments were performed in triplicate. 

### 2.15. Scratch Assay

At 24 h prior to transfection, 1 × 10^5^ T24 and MDA-MB 231 cells per well were seeded in a 6-well plate in 2000 µL of growth medium. The medium was replaced by 800 µL fresh medium and 200 µL MSN160 nm-454-GE11 (500 µg mL^−1^ in HEPES buffer) loaded with Ctrl RNA or miR200c was added. Cells were incubated with MSN160 nm-454-GE11 for 4 h, then the medium was changed. After 24 h additional incubation time, the cell layer was broken by a scratch using a 200 µL Eppendorf tip. Cells were washed with PBS and microscope (Axiovert 200, Zeiss, Oberkochen, Germany) pictures were taken after 24 h.

## 3. Results

### 3.1. Particle Synthesis

We synthesized a series of core-shell MSNs with uniform size, ranging from 60 to 160 nm via a delayed co-condensation method [17]. The core of all MSNs was functionalized with 4.5 mol% APTES (with respect to total silica) carrying amino groups in order to create a positively charged inner void volume capable of encapsulating the negatively charged RNA by electrostatic interaction. The outer surface was functionalized with 2 mol% mercaptopropyl triethoxysilane (MPTES) carrying a thiol functionalization to act as regiospecific linker for external binding of polymer and targeting ligand. 

Two slightly different approaches were used to modulate the particle size of MSNs and to prepare a series of MSNs with uniform sizes over a range from 60 to 160 nm. Larger particles with average particle sizes of 160, 130 and 100 nm (MSN160 nm, MSN130 nm and MSN100 nm) were synthesized based on our previous reports using a decreasing amount of the base triethanolamine (TEA) in order to reduce the particle size (experimental details are described in the Materials and Methods section) [43,44,46]. Briefly, to prepare the core of our core-shell particles, a preheated solution containing cetyltrimethylammonium chloride (CTAC) as template and triisopropylbenzene (TiPB) as pore-expanding agent was mixed with a preheated solution containing TEA, tetraethoxysilane (TEOS) and APTES at an elevated temperature for 20 min. This was then followed by addition of the ingredients for the surface layer containing TEOS and MPTES. To prepare smaller particles with average particle sizes of 80 and 60 nm (MSN80 nm and MSN60 nm), we applied a slightly different procedure, additionally using F127 as growth inhibitor/pore expanding agent according to published reports [45]. The particle size and core-shell structure were then established as above, again by changing the molar ratio of TEOS:TEA. Hereby, a preheated solution of octadecyltrimethylammonium bromide (C_18_Br) as template was mixed with a second preheated solution of TiPB, F127, TEA, TEOS and APTES, and stirred at 60 °C for 30 min. Afterwards, premixed TEOS and MPTES were added to form the negatively charged shell layer.

### 3.2. Characterization

**Figure 2:**
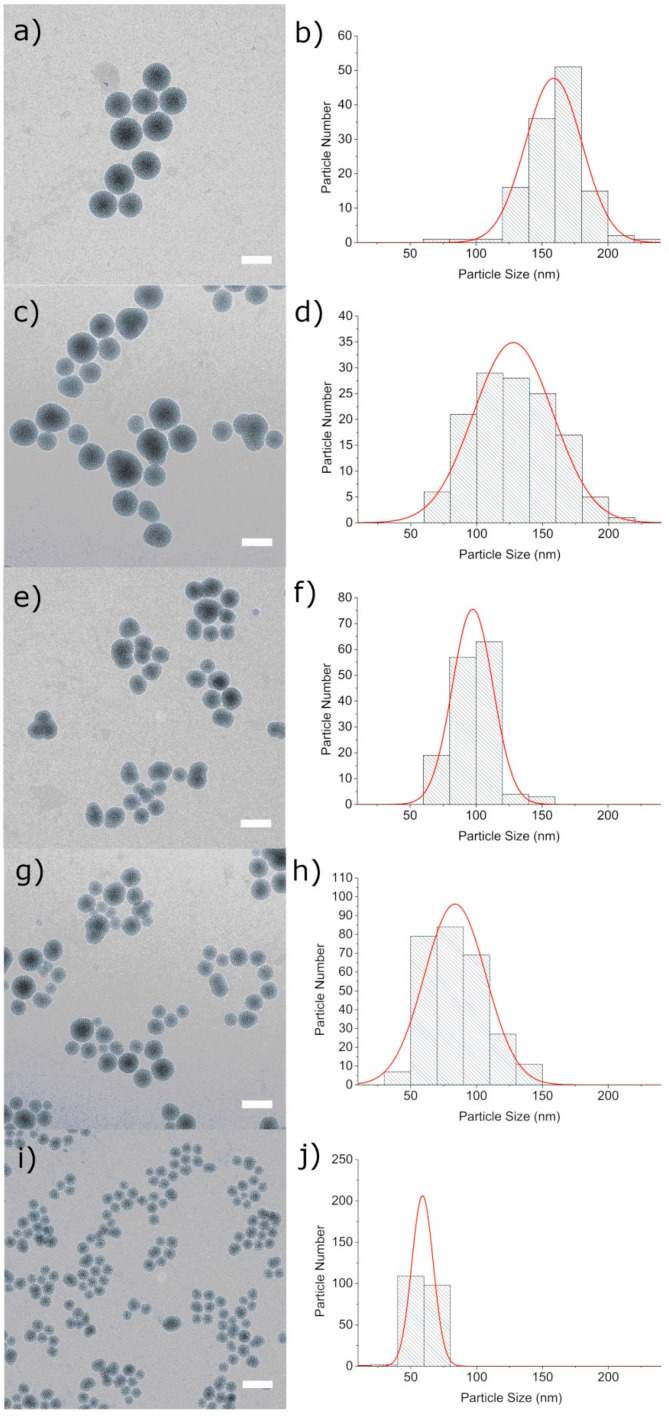
TEM micrographs of (**a**) MSN160 nm, (**c**) MSN130 nm, (**e**) MSN100 nm, (**g**) MSN80 nm, (**i**) MSN60 nm and corresponding particle size distribution histograms obtained from TEM images (**b**,**d**,**f**,**h**,**j**). Scale bar represents 150 nm.

The particles were characterized using a number of techniques including transmission electron microscopy (TEM), dynamic light scattering (DLS), thermogravimetric analysis (TGA), zeta potential measurements, Raman and IR spectroscopy. The particle size and the morphology were analyzed with TEM and DLS. All MSN samples show a spherical particle morphology with a disordered, wormlike pore structure, independent of size (Figure 2). Figure 2a,b shows a size distribution around 160 nm (sample MSN160 nm) for particles obtained with the CTAC synthesis using a molar ratio of TEOS:TEA = 1:10. As the molar ratio of TEOS:TEA was decreased to TEOS:TEA = 1:5 and 1:3, the average particle size decreased to 128 nm (sample MSN130 nm, Figure 2c,d) and 100 nm (sample MSN100 nm, Figure 2e,f), respectively. Particles obtained by the F127 synthesis show a mean particle size of 80 nm (sample MSN80 nm, TEOS:TEA = 1:5, Figure 2g,h). The smallest particles with a mean particle size of 60 nm (sample MSN60 nm, Figure 2i,j) were obtained with F127 and a TEOS:TEA ratio of 1:3. DLS measurements of suspended particles show slightly larger hydrodynamic diameters ranging from 90 to 250 nm (Figure S1) but clearly illustrate the same trend of a decreasing particle size with decreasing TEA concentration. 

**Figure 3:**
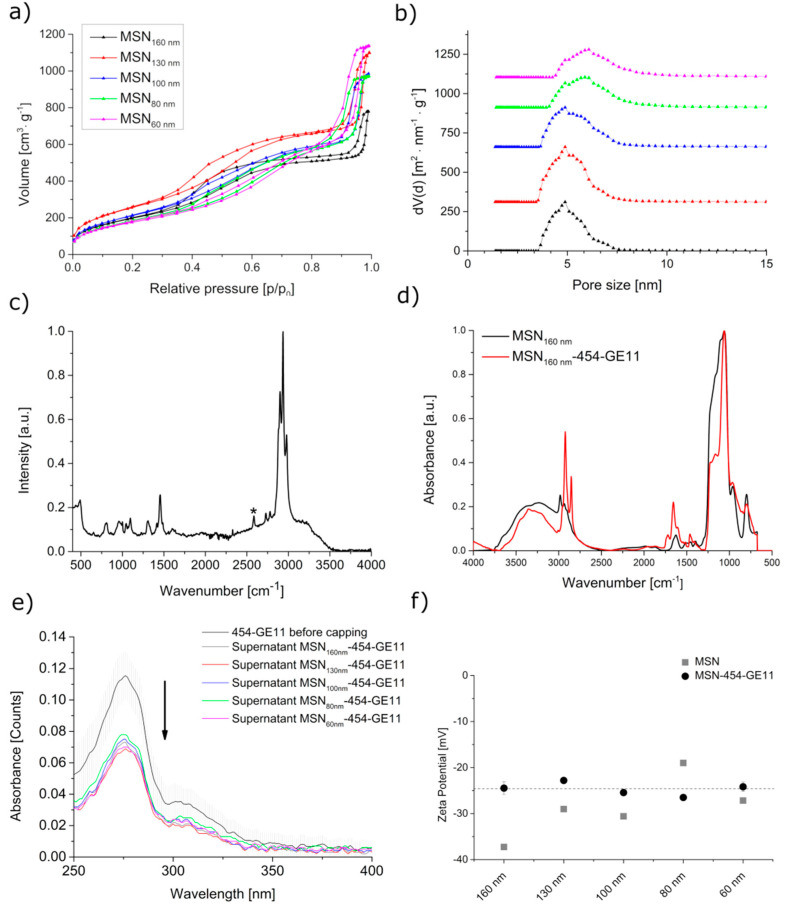
Characterization of MSN and MSN-454-GE11. (**a**) Nitrogen sorption isotherms and (**b**) corresponding pore size distributions. For clarity, the pore size distribution curves in panel b are shifted along the *y*-axis. (**c**) Raman spectrum of MSN160 nm. The signal at 2580 cm^−1^ (indicated by *) indicates the presence of thiol groups. (**d**) IR spectra, (**e**) UV VIS spectra of the capping solution before capping (black line) and the supernatants after capping, and (**f**) zeta potential measurements of MSN and MSN-454-GE11 at pH = 7.3.

Nitrogen sorption measurements resulted in typical type IV isotherms for all samples, as expected for MSNs (Figure 3a). Surface analysis indicates higher surface areas for particles prepared by the CTAC synthesis (samples MSN160 nm, MSN130 nm and MSN100 nm) ranging between 742–960 m^2^/g, and shows a similar pore size of about 4.8 nm. Slightly smaller surface areas but larger pore sizes were obtained for particles prepared by the F127 synthesis (MSN80 nm, MSN60 nm; 660–685 m^2^/g; pore size 6.0 nm, Figure 3b). Table 1 summarizes the properties of the particles obtained by the different synthesis methods. 

The presence of the amino and thiol groups originating from functionalization was verified with Raman and IR spectroscopy as well as with TGA. Representative results for sample MSN160 nm are shown in Figure 3c,d, respectively. Raman spectroscopy shows the S–H stretching mode of the thiol groups in the particle shell at 2580 cm^−1^ (Figure 3c), while the primary amines from core functionalization are seen at 1630 cm^−1^ with IR spectroscopy (MSN160 nm, black line, Figure 3d). TGA confirms the inclusion of organic functional groups by a weight loss of 20% (Figure S2). The decomposition of aminopropyl and successively, the mercaptopropyl groups starts at 290 °C.

### 3.3. Polymer Capping with 454-PEG and 454-GE11 and Targeting

The surface of the MSNs was capped with a modularly designed block copolymer 454 to prevent a premature release of the cargo and to enhance endosomal escape for intracellular delivery of miRNA. The structure of the copolymer 454 is shown in Figure S3. This T-shaped polymer 454 consists of a hydrophobic domain in the center made of two oleic acids attached to lysine units. Branched off at each side from the center are two succinyl-tetraethyl-pentamine (Stp) units [47], providing the polymer with a cationic charge. Each end contains a tyrosine trimer coupled to a terminal cysteine unit, potentially allowing additional functionalization. The combination of the central Stp and oleic acid units in 454 is assumed to facilitate interactions with the endosomal membrane, resulting in endosomal destabilization and thus enabling the nucleic acid cytosolic delivery. This block copolymer was successfully used for the formulation of INF-7-polyplex/siRNA vehicles [48] and was further essential for the highly efficient delivery of siRNA using MSNs, reported previously [21].

An anticancer therapy can potentially be improved by implementing cancer-cell-specific targeting molecules to the external surface of a carrier system. It is well known that EGF receptors (EGFR) are concentrated on many cancer cells. The small peptide GE11 has been used successfully before to specifically address EGFR-expressing cells [41,49,50]. Here, we exploit the mercapto residues of the cysteine groups in the capping polymer 454 for binding GE11 via a PEG linker to study the targeted, particle-size-dependent delivery of the miR200c.

For anchoring GE11 to the cysteine units in the block copolymer, we used a mercapto-reactive maleimide-PEG moiety consisting of 28 ethylene glycol monomer units that was previously linked to the GE11 ligand (Mal-PEG-GE11 reagent). The Mal-PEG-GE11 reagent was then mixed with the 454 block-copolymer (454-GE11). Only 0.1 equivalents (eq.; relative to 454-polymer) of the targeting ligand were used for 454-functionalization to maintain free thiol groups at the end of 454. The latter can potentially undergo a disulfide bridging with the terminal mercapto-groups on the outer surface of the MSNs, thus creating a stable copolymer capping. The attachment of the cationic polymer 454 to the surface is additionally electrostatically favored since zeta potential measurements of our pure MSNs reveal a negative surface charge for all samples at a pH higher than 5.5 (Figure S4). To perform the attachment of the premixed 454-GE11 to the surface of MSN, a suspension of MSN at pH 7.3 was mixed with 454-GE11 and the successful capping was confirmed using UV–VIS, IR, and zeta potential measurements. IR spectroscopy shows a significant increase of the CH stretching vibrations between 2850 and 2930 cm^−1^ and of the C-H bending vibrations of 1460 cm^−1^ as compared to the bare MSN particles (red graph in Figure 3d). Furthermore, the MSN-454-GE11 spectrum shows an increase of the N–H bending vibrations at 1650 cm^−1^ and a new signal at 1720 cm^−1^ related to a C=O stretching mode, indicating the multiple amide bonds of the copolymer. UV–VIS measurements were further used to quantify the amount of the 454-GE11 capping agent, which was similar in all samples and ranged from 17 to 20 wt% (Figure 3e, calibration curve Figure S5 and capping amount of different samples, Table S1).

Figure 3f shows the zeta potential measurements of all MSN samples before and after attachment of the 454-GE11 linker (MSN-454-GE11) at pH = 7.3. Besides the size of the nanoparticles, their surface charge is an important parameter for cellular internalization. Our pure MSN samples display a negative surface charge between −37 and −20 mV, as expected for mercapto-covered MSNs. The zeta potential of all capped MSN-454-GE11 samples is very comparable and has increased to about −24 mV when measured at pH 7.3. The observed difference before and after capping can be seen as an additional indication for a successful attachment of the targeted copolymer. The polymer capping did not affect the particle size distribution in water for most of the MSN-454-GE11 samples when measured by DLS. Only the sample with a mean particle size of 60 nm showed some moderate agglomeration (Figure S6).

### 3.4. Cell Internalization

As documented above, we have now established that our MSN carrier system shows physical properties such as surface area, pore size, zeta potential and capping concentration that are all very comparable, thus, leaving solely the particle size as a distinct variable. First, flow cytometry and confocal fluorescence microscopy data were used to investigate how the targeting ligand concentration as well as particle size might influence the cell internalization of the fully assembled MSN-454-GE11 samples.

**Figure 4:**
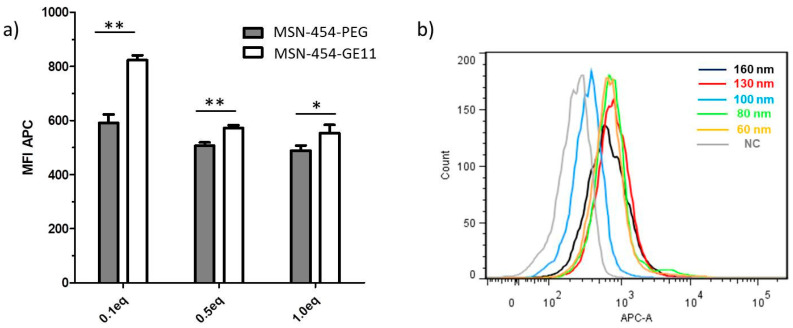
Cellular internalization determined by flow cytometry. Cells were incubated for 45 min, washed and analyzed. (**a**) MSN160 nm-454-PEG was used for passive and MSN160 nm-454-GE11 for receptor-mediated uptake. 454 was functionalized with different equivalents of Mal-PEG (454-PEG) and Mal-PEG-GE11 reagent (454-GE11), respectively, in the range from 0.1 to 1 eq. The mean fluorescence intensity of the Atto-633 signal (MFI APC) represents the amount of internalized nanoparticles. A high MFI corresponds to a large cell uptake of MSN. For statistical analysis, a two-tailed t-test was performed (*n* = 3, mean ± SD, * *p* < 0.05, ** *p* < 0.01) (**b**) Histograms of cellular internalization of Atto-633 labeled MSN-454-GE11 after 45 min incubation with particle sizes in the range of 160 nm to 60 nm and negative control (NC). The Atto-633 intensity (APC-A channel) is plotted against the number of events detected (‘count’). ‘Count’ represents cumulative counts of cells with indicated Atto-633 fluorescence after appropriate gating by forward/sideward scatter and pulse width. For statistical analysis, see Figure S7.

Figure 4 shows the cellular internalization of the MSNs by T24 cells as examined via flow cytometry after a 45 min incubation time. MSN-454-GE11 samples were labeled with the Atto-633-carboxy fluorescent dye, which was covalently bound to the amino groups in the core of the particles. In order to investigate the best targeting concentration, we mixed different weight equivalents of targeting ligand GE11 with the 454 polymer in the range from 0.1 to 1 eq. before attaching the polymer to the MSN samples. Maleimide-PEG without ligand was used as negative control for polymer capping without targeting ligand (sample MSN-454-PEG). A significant targeting effect can be seen in Figure 4a when 0.1 eq. of the targeting ligand was used, while only a minor improvement is observed at higher concentrations. 

**Figure 5:**
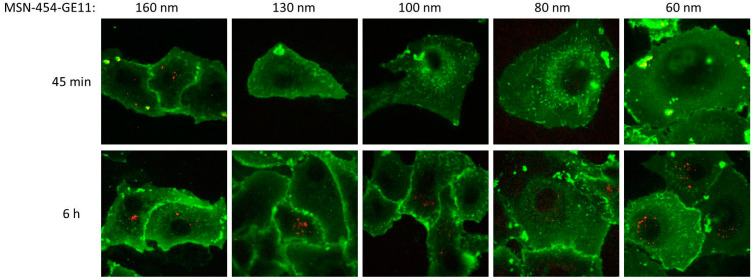
Representative confocal fluorescence microscopy images of Atto-633 labeled MSN-454-GE11 (red) with particle sizes in the range of 160 nm to 60 nm after 45 min (upper panel) and 6 h (lower panel) incubation on WGA488-stained T24 cells (green). For a statistical evaluation of particle-size-dependent uptake, see Figure S9, SI.

Figure 4b documents the particle-size-dependent association of MSN-454-GE11 to cells as investigated via flow cytometry. Flow cytometry showed a similar degree of cell association for all samples, while a slight trend towards stronger association can be seen for smaller particles (quantification and statistical analysis can be found in SI, Figure S7). Since flow cytometry is not able to differentiate internalization from externally adhering particles, we followed this process also with confocal fluorescence microscopy as shown in Figure 5 (for enlarged images with additional orthogonal views of each image showing the particle internalization, see Figure S8, Supplementary Materials). Images were subsequently analyzed via the digital method ‘Particle_in_Cell-3D’ to quantify the cellular uptake of the differently sized MSN vectors. After imaging cells directly after an incubation time of 45 min, we found that only MSN160 nm-454-GE11 particles had penetrated through the cell membrane and were truly internalized. Other smaller MSN-454-GE11 particles were only attached to the outer cell membrane at this time. In contrast, all particle sizes were internalized after a 6 h incubation time. However, also after this time, MSN160 nm-454-GE11 particles showed the highest number of internalized particles of all samples (Figure S9).

### 3.5. Loading of RNA

Loading of the genetic material into our MSN samples was performed with all samples prior to the attachment of the block copolymer. The adsorption of the miRNA is mainly driven by electrostatic interactions with the cationic, amino-functionalized core of the MSNs. The negatively charged thiol groups at the particle periphery are expected to minimize any external adsorption. For the best results, we performed the RNA adsorption in MES buffer at pH = 5. The MSN particles show a positive zeta potential at this pH due to protonation of the amino groups (zeta potential titration, see Figure S4).

**Figure 6:**
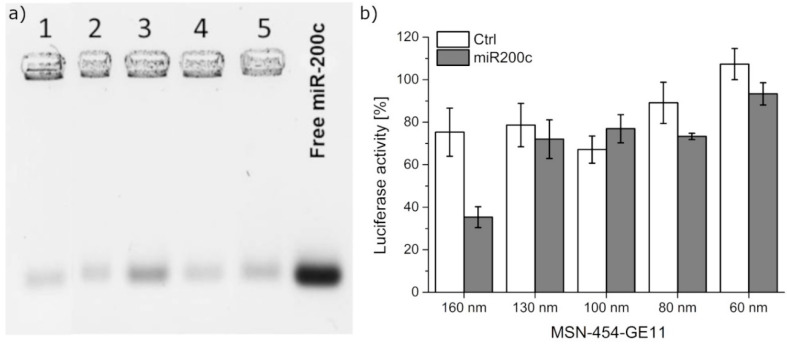
(**a**) Gel electrophoresis of samples MSN160 nm-454-GE11 (1), MSN130 nm-454-GE11 (2), MSN100 nm-454-GE11 (3), MSN80 nm-454-GE11 (4), and MSN60 nm-454-GE11 (5). (**b**) Gene silencing of T24/eGFPLuc-200cT cells transfected with MSN-454-GE11 containing either Ctrl (white) or miR200c (grey). After an incubation time of 45 min, cells were washed. At 48 h after transfection, gene-silencing effects were analyzed.

Here, aliquots of 50 µg mg^−1^ RNA/MSN were used for loading and the actual uptake was calculated by difference measurements by determining the remaining RNA concentration in the supernatant. All samples were able to adsorb the amount offered, as no residual RNA could be detected in the supernatant after loading times as short as 30 min. The samples were subsequently capped with 454-GE11 to obtain the final MSN-454-GE11 samples used for subsequent experiments. The stable binding of RNA in these samples is reflected in gel shift results, as shown in Figure 6a). Only little RNA elution is visible upon applying a voltage of 100 V for 1 h to the gel.

### 3.6. Gene Silencing

As a proof of concept, the gene silencing of the eGFP-luciferase reporter gene was performed using T24/eGFPLuc-200cT cells. This cell line stably expresses the eGFP-luciferase fusion protein, while simultaneously featuring a miR200c target site on the expressed mRNA. Therefore, the gene expression of eGFP-luciferase fusion protein can be blocked by the delivery of miR200c. The luciferase signal was thus used to evaluate the gene-silencing efficacy of miR200c. In order to simulate the dynamic conditions of drug delivery and to avoid a prolonged overexposure of the cells with particles suspended in the medium, we have used extremely short incubation times of 45 min. Cells were subsequently washed by exchanging the medium and the effect of this short exposure was then analyzed after time spans referred to as transfection time in the following. Cells were transfected with MSN and MSN-454-GE11 of different sizes loaded with either a synthetic miR200c mimic or a control siRNA without any target gene (Ctrl, Figure 6b). Gene silencing was never observed with the pure MSN samples (missing the 454 polymer construct) loaded with miR200c for silencing (Figure S10a). This might be caused by a premature release of the RNA or by endosomal trapping of the MSN samples missing the 454 polymer. 

Similarly, none of the smaller sized MSN-454-GE11 samples showed any significant silencing efficacy. In contrast, with the larger 160 nm targeted polymer-capped MSN160 nm-454-GE11 sample, a luciferase gene knockdown of up to 65% was observed. These findings are in good agreement with the confocal fluorescence microscopy images, which showed the fastest internalization for particles with a size of 160 nm. 

Flow cytometry data showed an improved cellular internalization of the targeted MSN160 nm-454-GE11 vector in comparison with the non-targeted control MSN-454-PEG when 0.1 eq. of the targeting ligand was used. This targeted vector showed a higher silencing efficacy compared to the non-targeted control, which is in good agreement with the increased uptake observed via flow cytometry.

As expected, the same sample loaded with just scrambled RNA (Ctrl) did not show a significant unspecific knockdown, thus excluding major toxic effects induced by the carrier system itself. Systematic cell viability studies using an MTT assay also show a good tolerance of the cells towards these samples (Figure S11).

These results show that a particle size of about 160 nm in combination with the copolymer 454 enables the fastest cell internalization and also the best transfection efficacy when short incubation times of only 45 min are used. Inhibition experiments addressing specific endocytic pathways were inconclusive with respect to a preferred mechanism for a certain particle size. However, endocytosis was substantially more blocked for the larger particles as compared to the 60 nm particles (see Figure S12, Supplementary Materials). Our data also confirm the endosomolytic activity of the 454 polymer since cell adherence/uptake was observed for all pure MSN samples (flow cytometry of pure MSN samples in Figure S13), but no transfection occurred without the 454 polymer (see Figure S10a). As described above, we further found that only MSN160 nm-454-GE11 showed gene-silencing activity, while all smaller MSN-454-GE11 samples were not active after the short incubation period applied here. Confocal fluorescence microscopy showed that MSN160 nm-454-GE11 particles were already internalized after 45 min, while all other MSN-454-GE11 samples needed much longer incubation times to achieve internalization. Thus, particle internalization of the smaller particles is likely too slow and prevents a larger efficacy.

Recently, miR200c was delivered by some of us using GE11 modified 454 polyplexes with a similar size of 120–150 nm [41]. The gene silencing efficiency achieved in the same T24/eGFPLuc-200cT cells with these polyplexes was around 60%, which is in the same range as the knockdown efficiency of the MSN-454-GE11 constructs presented here. This comparison suggests that the size of the targeted constructs is a determining feature controlling the knockdown efficiency. As MSN160 nm-454-GE11 constructs were shown to offer the best cellular-uptake behavior and gene-silencing efficiency, they were used for the following cell migration and cell cycle experiments. 

### 3.7. Antitumoral Effects

The tumor suppressor miR200c inhibits the epithelial–mesenchymal transition, a process involved in metastasis by enhancing the motility and migration of tumor cells, by targeting ZEB1 and ZEB2. ZEB1 and ZEB2 are transcriptional repressors, which downregulate the marker E-cadherin [7,51]. Furthermore, Kopp et al. reported that one of the most prominent oncogenes KRAS is targeted by miR200c, which results in an altered cell cycle of the cancer cells [6]. Notably, they could show that miR200c inhibits cell cycle progression by decreasing the G1-population. We have performed direct investigations of these antitumoral effects through tumor cell migration and cell cycle analysis on two different EGFR overexpressing cell lines, T24 bladder cancer and HeLa cervical cancer cells using miR200c loaded MSN-454-GE11 samples. 

**Figure 7:**
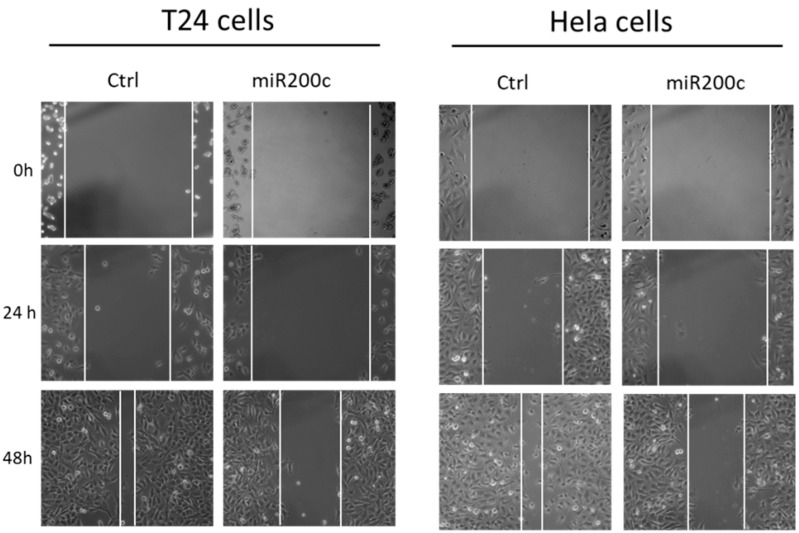
Inhibition of tumor cell migration. Images of a scratch assay of T24 and HeLa cells. Cells were treated with MSN160 nm-454-GE11 loaded with miR200c. The cell layer was broken after 24 h through a scratch and the closure was monitored for 48 h.

Cell migration was studied using a scratch assay, as shown in Figure 7. T24 cells and HeLa cells were incubated with MSN-454-GE11 for 4 h (5 µg miR200c/well), then the medium was changed. After additional 24 h, the cell layer was broken by a scratch using a 200 µL Eppendorf pipette tip. The closure of the scratch, which is an indicator for cell migration, was measured at indicated time points. In both cell lines, the scratch was almost completely closed after 48 h when MSN-454-GE11 was loaded with Ctrl-RNA (88% scratch closure for T24 and 82% closure for HeLa cells). In contrast, with MSN-454-GE11 particles loaded with miR200c, a scratch closure of only 48% (for T24) and 53% (for HeLa cells) was observed after this time. Hence, miR200c delivered by MSN-454-GE11 significantly hinders cell migration.

**Figure 8:**
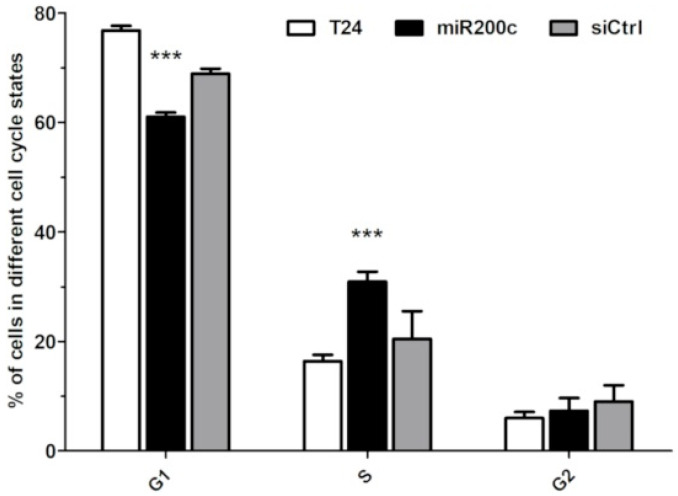
Cell cycle analysis via flow cytometry of cell stages G1, S and G2 of T24 cells at 72 h after treatment. For statistical analysis, a two-tailed t-test was performed (*n* = 3, mean ± SD, *** *p* < 0.01)

In addition, the effect of miR200c on the cell cycle was studied. Cells were transfected with MSN-454-GE11 loaded with miR200c or with the control RNA Ctrl and incubated for 4 h (Figure 8). A significant decrease in the number of cells in the G1 phase is observed when exposed to miR200C delivered by MSN, in combination with an increase in the number of cells in the S-phase. Thus, tumor cells transfected with MSN-454-GE11 loaded with miR200c showed the expected decreased migration and changes in the cell cycle. 

## 4. Conclusions

In this study, we exploited the tunability of MSNs to synthesize a series of core-shell MSNs with different particle sizes between 60 to 160 nm for studying the size effect of these carriers on antitumoral miRNA delivery. All other properties of the delivery vehicles, including surface area, pore size and zeta potential were kept comparable. The nanoparticles were capped with a positively charged block copolymer 454 equipped with the targeting agent Mal-PEG-GE11. Since gene silencing was only observed after capping the nanocarriers with this 454 polymer, we conclude that it is essential for the endosomal escape by destabilizing the endosomal membrane. It was shown that the targeting ligand GE11 enhances a receptor-mediated uptake. After capping, the MSN-454-GE11 vehicles were used for a systematic investigation of size-dependent gene silencing. While smaller particles did not lead to significant effects, MSN-454-GE11 with a size of 160 nm showed a remarkable gene knockdown efficacy and antitumoral effects such as a decreased migration and changes in cell cycle. Overall, we observed the fastest cellular internalization as well as the best knock-down efficacies with MSN-454-GE11 sized 160 nm. In contrast to FACS results that indicated a particle association with the cells independent of MSN particle size, we found with statistically evaluated image analysis that only the largest particles are truly internalized in the cells after short incubation times. Thus, cell studies as performed here, aiming to simulate dynamic conditions of in vivo drug delivery by washing the incubated cells after a short incubation time, might discriminate against all particles that are not well attached to the cell surface. Fast internalized particles are thus the winner. We hypothesize that due to their size, they expose a larger contact area to the cell membrane and simultaneously a larger number of targeting ligands, which, when spaced just right, allow for a maximum degree of endocytosis. Our study shows that fast cellular internalization is essential for a successful downregulation. In summary, the nanoscale MSN160 nm-454-GE11 vehicles show the most promising potential for future in vivo biomedical applications.

## Figures and Tables

**Table 1:** Synthesis and properties of MSN samples.

Name	Synthesis Method	Diameter TEM ^a^ [nm]	A_BET_ [m^2^/g]	Pore Volume ^b^ [cc/g]	Pore Size ^c^ [nm]
MSN160 nm	CTAC Synthesis; TEOS:TEA = 1:10	159 ± 21	742	0.80	4.8
MSN130 nm	CTAC Synthesis; TEOS:TEA = 1:5	128 ± 30	961	1.06	4.8
MSN100 nm	CTAC Synthesis; TEOS:TEA = 1:3	100 ± 37	811	0.95	4.8
MSN80 nm	F127 Synthesis; TEOS:TEA = 1:5	84 ± 23	685	0.95	6.0
MSN60 nm	F127 Synthesis; TEOS:TEA = 1:3	59 ± 8	660	1.00	6.0

^a^ The average particle size was obtained from TEM micrographs by measuring the diameter of around 200 particles of respective samples. ^b^ The total pore volume was determined at *p*/*p*_0_ = 0.9 to exclude contributions of textural porosity. ^c^ Data were acquired from the adsorption branch of the nitrogen isotherm.

## Supplementary Materials

The following are available online at https://www.mdpi.com/1999-4923/12/6/505/s1, Figure S1: DLS measurements of MSN samples, Figure S2: TGA, Figure S3: Structure of copolymer 454, Figure S4: Zeta potential titration curves of pure MSN samples, Figure S5: 454-GE11 calibration curve, Figure S6: DLS measurements of MSN-454-GE11 samples, Figure S7: Quantification of FACS data shown in Figure 4b, Figure S8: Confocal fluorescence microscopy images, Figure S9: Quantification of the cellular uptake of MSN-454-GE11, Figure S10: Gene-silencing assay, Figure S11: MTT cell viability study, Figure S12: Inhibition results, Figure S13: FACS of internalization of untargeted nanoparticles, Table S1: Estimated capping amount of 454-GE11 for different MSN samples.
