# Supplementary material for: Particle-Size-Dependent Delivery of Antitumoral miRNA Using Targeted Mesoporous Silica Nanoparticles
Source: Pharmaceutics. 2020 Jun 2;12(6):505. doi: 10.3390/pharmaceutics12060505 (PMC7355705; doi:10.3390/pharmaceutics12060505)
Supplement: Supplementary file 1 [file pharmaceutics-12-00505-s001.pdf]

pharmaceutics

Supplementary Materials: Particle-Size-Dependent Delivery of Antitumoral miRNA using Targeted Mesoporous Silica Nanoparticles

Lisa Haddick, Wei Zhang, Sören Reinhard, Karin Möller, Hanna Engelke, Ernst Wagner and Thomas Bein *

DLS Measurements of MSN Samples

The particles obtained via the reported synthesis procedure were characterized using DLS. The different samples show an average hydrodynamic radius between 90 and 250 nm.

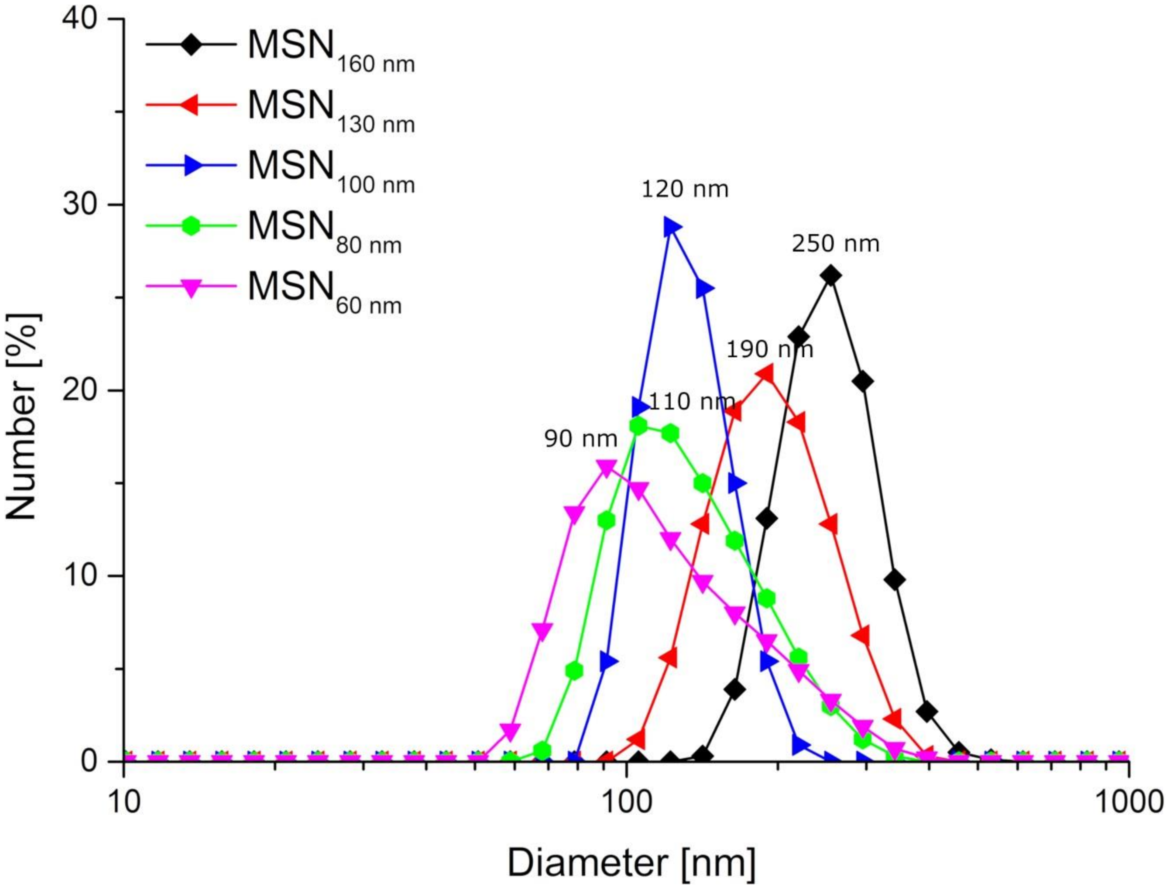

Figure S1. DLS measurements of MSN samples in EtOH: MSN_{160nm} (black line), MSN_{130nm} (red line), MSN_{100nm} (blue line), MSN_{80nm} (green line), and MSN_{60nm} (magenta line).

TGA Data of MSN160 nm up to 900 °C

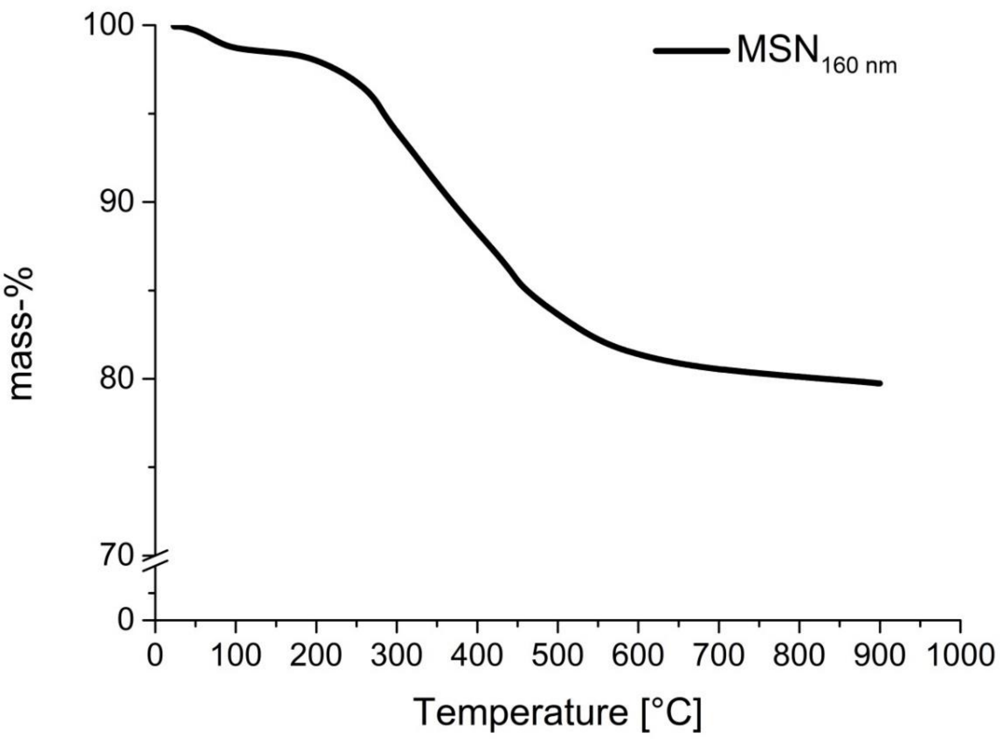

Figure S2. TGA curves of sample MSN_{160nm} measured up to 900 °C.

Structure of Copolymer 454

The structure of the copolymer is depicted in Figure S2.

454

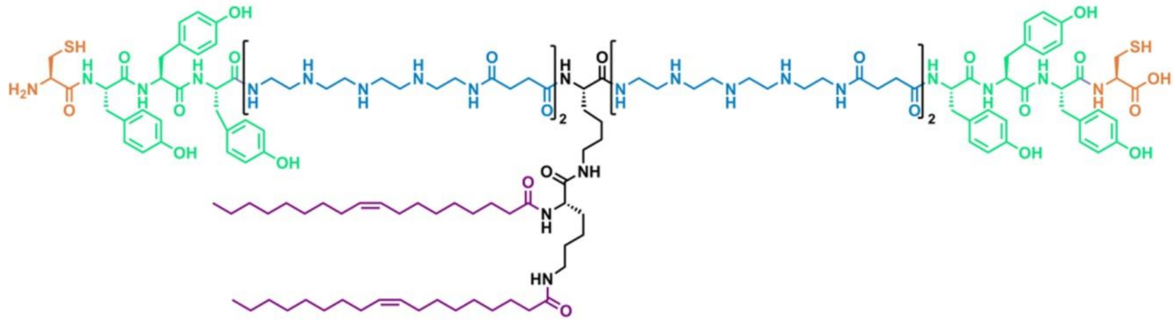

Figure S3. Structure of copolymer 454.

Zeta Potential of MSN Samples

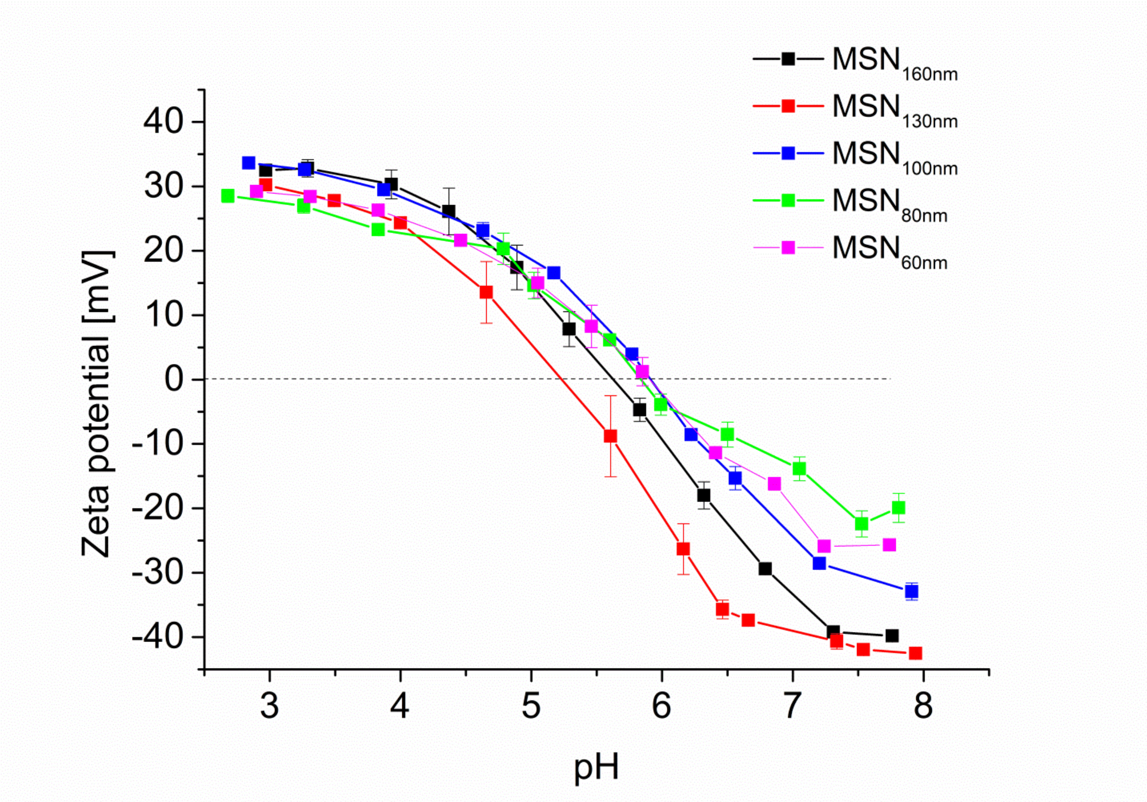

Figure S4. Zeta potential titration curves of pure MSN samples: MSN_{160nm} (black line), MSN_{130nm} (red line), MSN_{100nm} (blue line), MSN_{80nm} (green line), and MSN_{60nm} (magenta line).

UV-VIS Measurements for Quantification of Capping Amount of 454-GE11

The amount of the attached 454-GE11 polymer was obtained by difference measurements: increasing concentrations of 454-GE11 in MES buffer were used to obtain a calibration curve and were measured at a spectral maximum of 275 nm. For determining the capping concentration, we added a 454-GE11 solution to a defined amount of MSN in MES buffer. After 2 h, the particles were centrifuged to form a pellet and the unreacted 454-GE11 in the supernatant was determined by UV-VIS spectroscopy. A substantial decrease in absorbance in the coupling solution was observed for all MSN samples. The particles were capped with 454-GE11 with amounts ranging between around 17–20 wt% (detailed amounts are listed in Table S1).

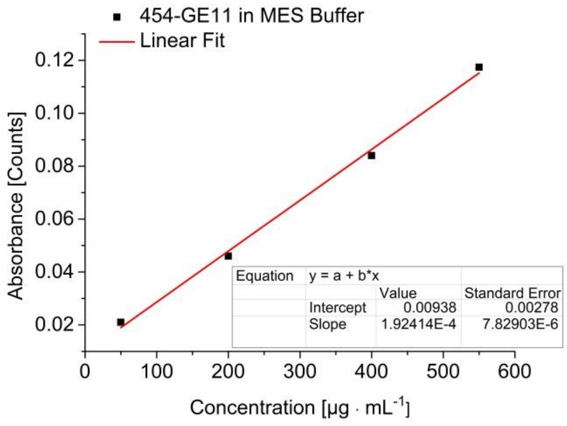

Figure S5. 454-GE11 calibration curve measured at 275 nm for quantification of the capping concentration.

Table S1. Estimated capping amount of 454-GE11 for different MSN samples.

MSN-454-GE11	Difference Concentration before and after Capping [µg/mL]	Capping Amount wt%
160 nm	230,7	18,7
130 nm	256,7	20,4
100 nm	220,3	18,0
80 nm	204,7	17,0
60 nm	246,3	19,7

Comparison of DLS Measurements of Pure MSN and MSN-454-GE11 Samples of Different Sizes

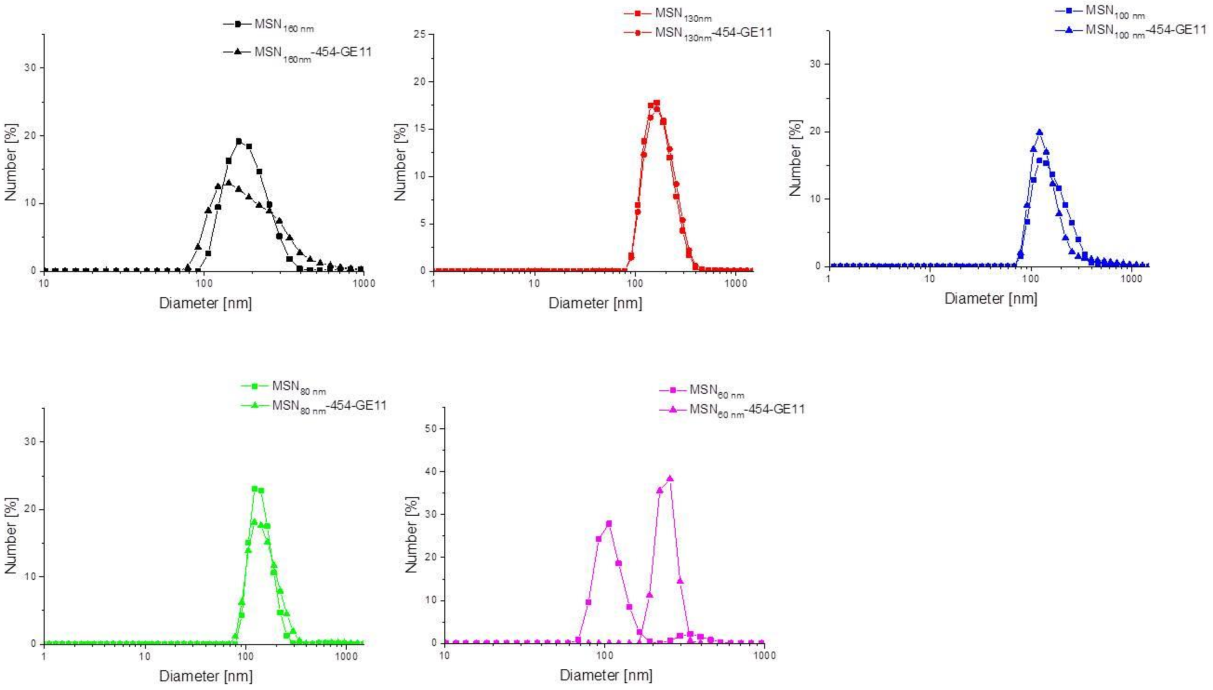

Figure S6. DLS measurements of MSN and corresponding MSN-454-GE11 samples in HEPES buffer: MSN_{160nm} (black line), MSN_{130nm} (red line), MSN_{100nm} (blue line), MSN_{80nm} (green line), MSN_{60nm} (magenta line).

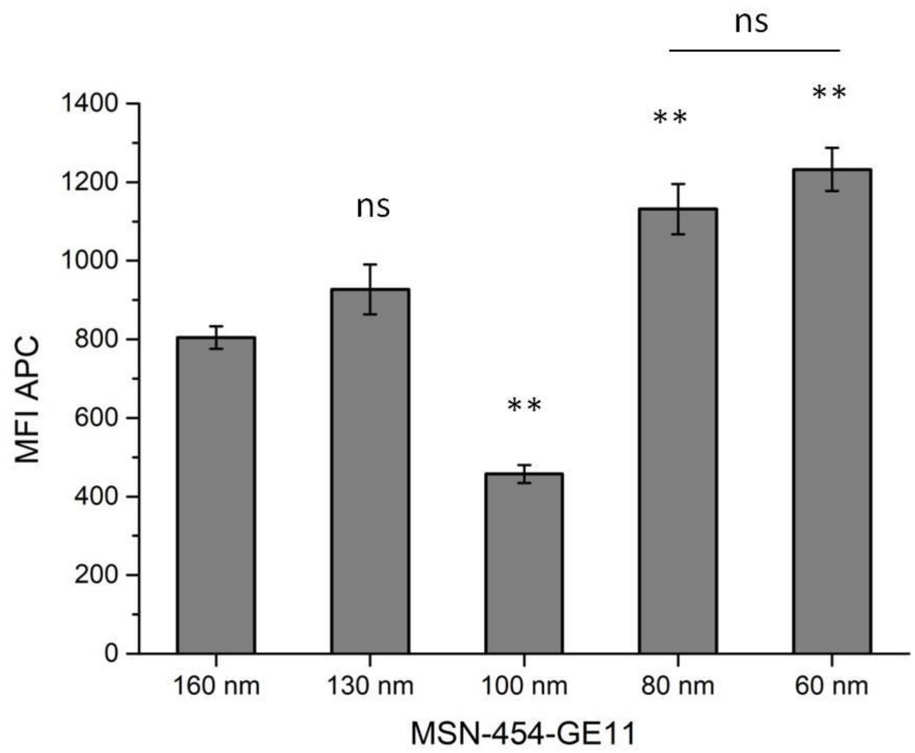

Figure S7. Quantification of the FACS data shown in Figure 4b in the main text. It shows the cellular internalization of Atto-633 labeled MSN-454-GE11 after 45 min incubation with particle sizes in the range of 160 nm to 60 nm. For statistical analysis a two-tailed t-test was performed ($n = 3$, mean ± SD, ns (not significant) $p > 0.05$, * $p < 0.05$, ** $p < 0.01$). Statistical significance indications on top of the flow cytometry bars without connecting line show statistical significance between 160 nm sample and other sizes of MSN-454-GE11 samples. Indicated statistical significance with connecting line shows no statistical significance between MSN80 nm-454-GE11 and MSN60 nm-454-GE11.

Confocal Microscopy including Orthogonal View

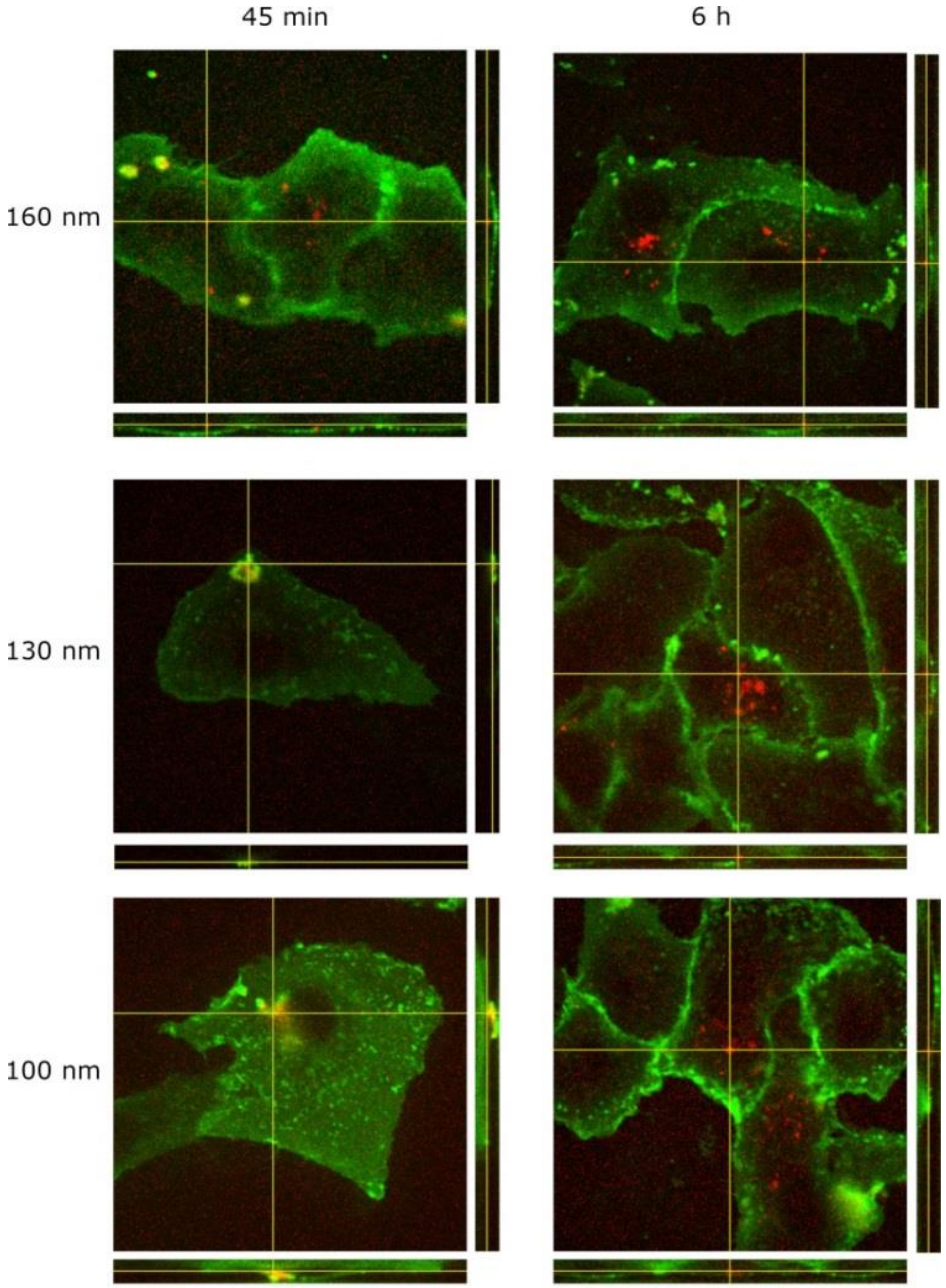

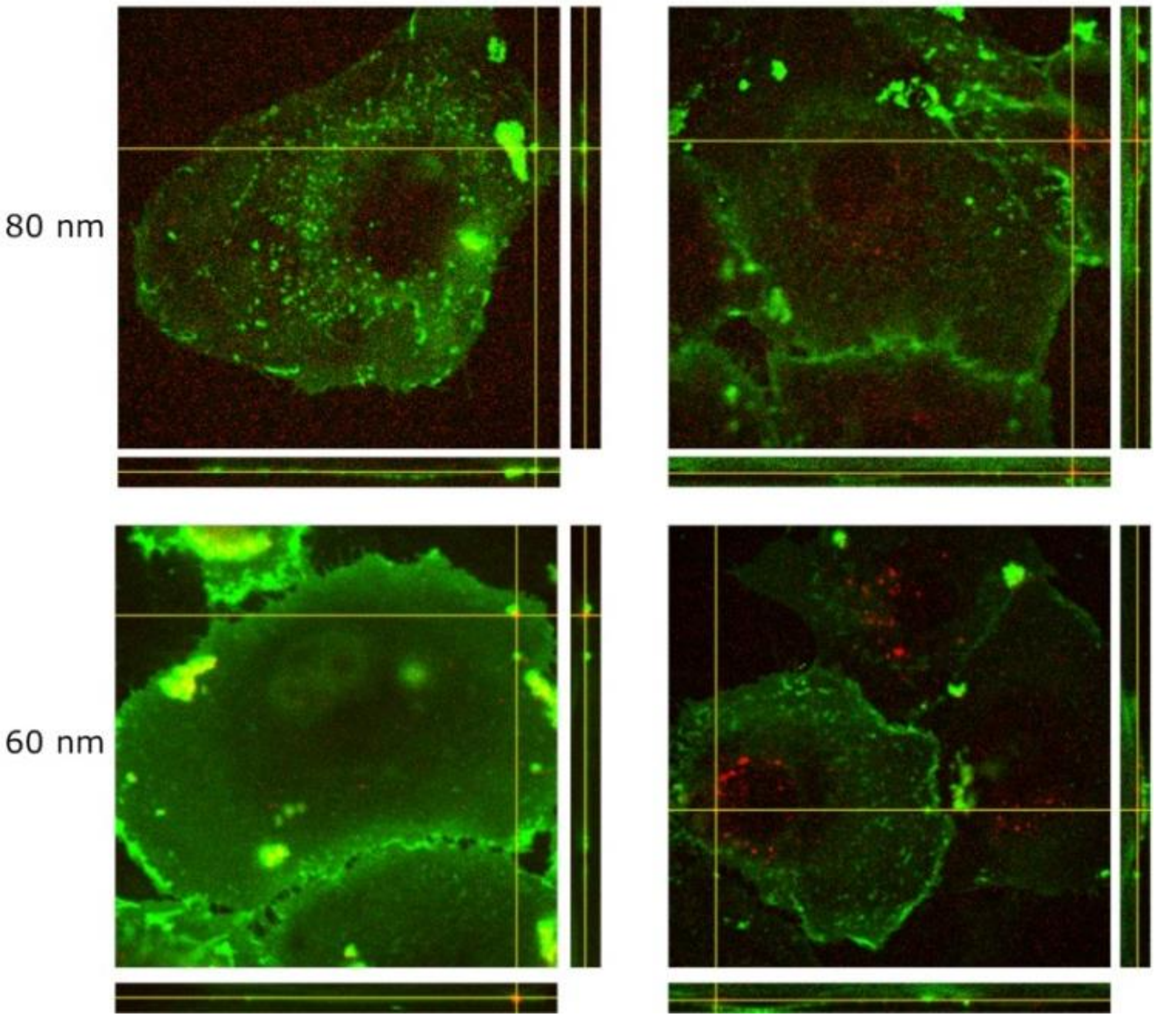

Figure S8. Enlarged representative confocal fluorescence microscopy images of Atto-633 labeled MSN-454-GE11 samples (red) with particle sizes in the range of 160 nm to 60 nm after 45 min and 6 h of incubation on WGA488-stained T24 cells (green), respectively. The orthogonal views on the side of each image show the particle internalization.

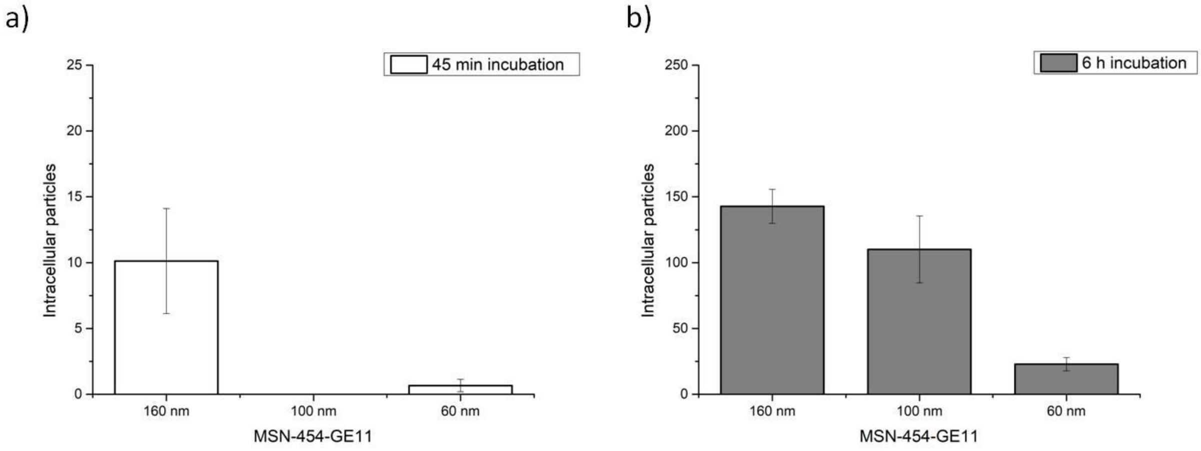

Figure S9. Quantification of the cellular uptake of MSN-454-GE11 with 160, 100 and 60 nm particle sizes in T24 cells after a) 45 min and b) 6 h incubation time using Particle_in_Cell-3D as analysis method for confocal fluorescence microscopy images. The histograms represent three independent experiments (n = 21–25).

Gene Silencing of eGFP-Luciferase using Pure MSN Samples without 454-GE11

No silencing efficacy was observed when the gene silencing of the eGFP-luciferase reporter gene was examined using pure MSN samples without the capping/targeting ligand 454-GE11 but loaded with either miR200c or siCtrl RNA (Figure S11a). Figure 11b shows the comparison of silencing efficacy of the targeted MSN160 nm-454-GE11 sample to a non-targeted control (MSN-454-PEG). The non-targeted MSN show again a lower effectivity, as was anticipated from the flow cytometry results shown in Figure 4a.

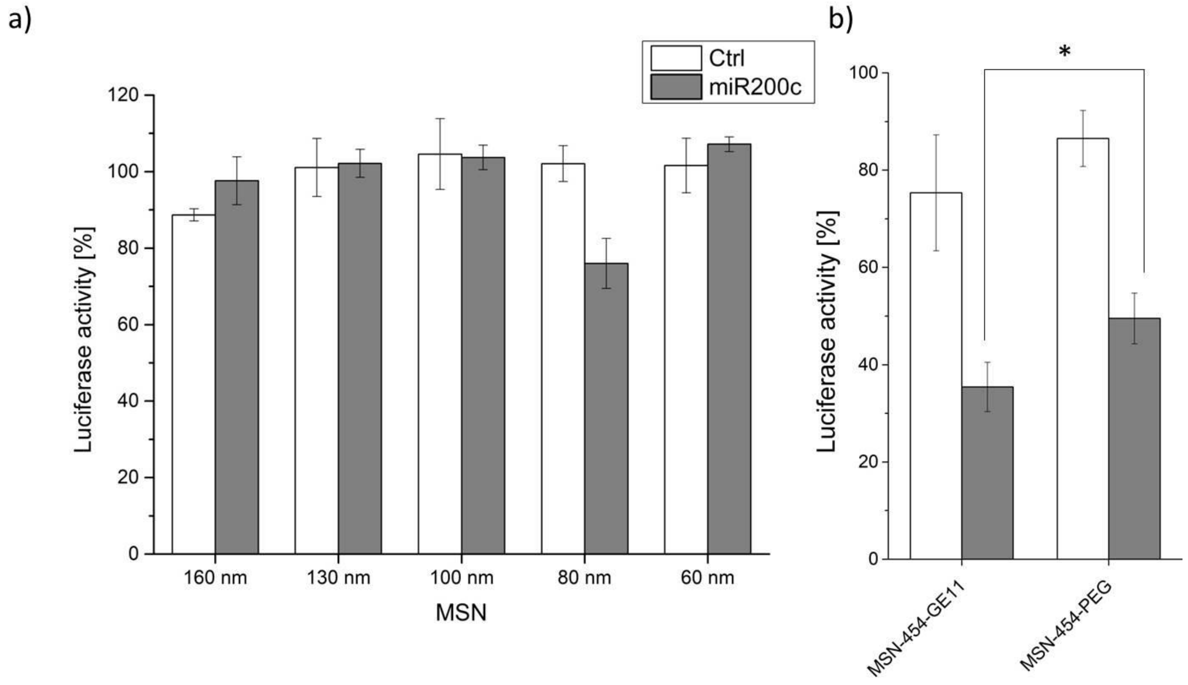

Figure S10. Gene-silencing assay using a) pure MSN loaded with miR200c or Ctrl, but without the capping/targeting ligand 454-GE11, b) MSN 160 nm-454-GE11 as a targeted sample in comparison to a non-targeted MSN160 nm-454-PEG sample. For statistical analysis a two-tailed t-test was performed (n = 3, mean ± SD, * p < 0.05). All samples were incubated for 45 minutes and transfected for 48 h after medium change.

Cell Viability Assay with Sirna Control

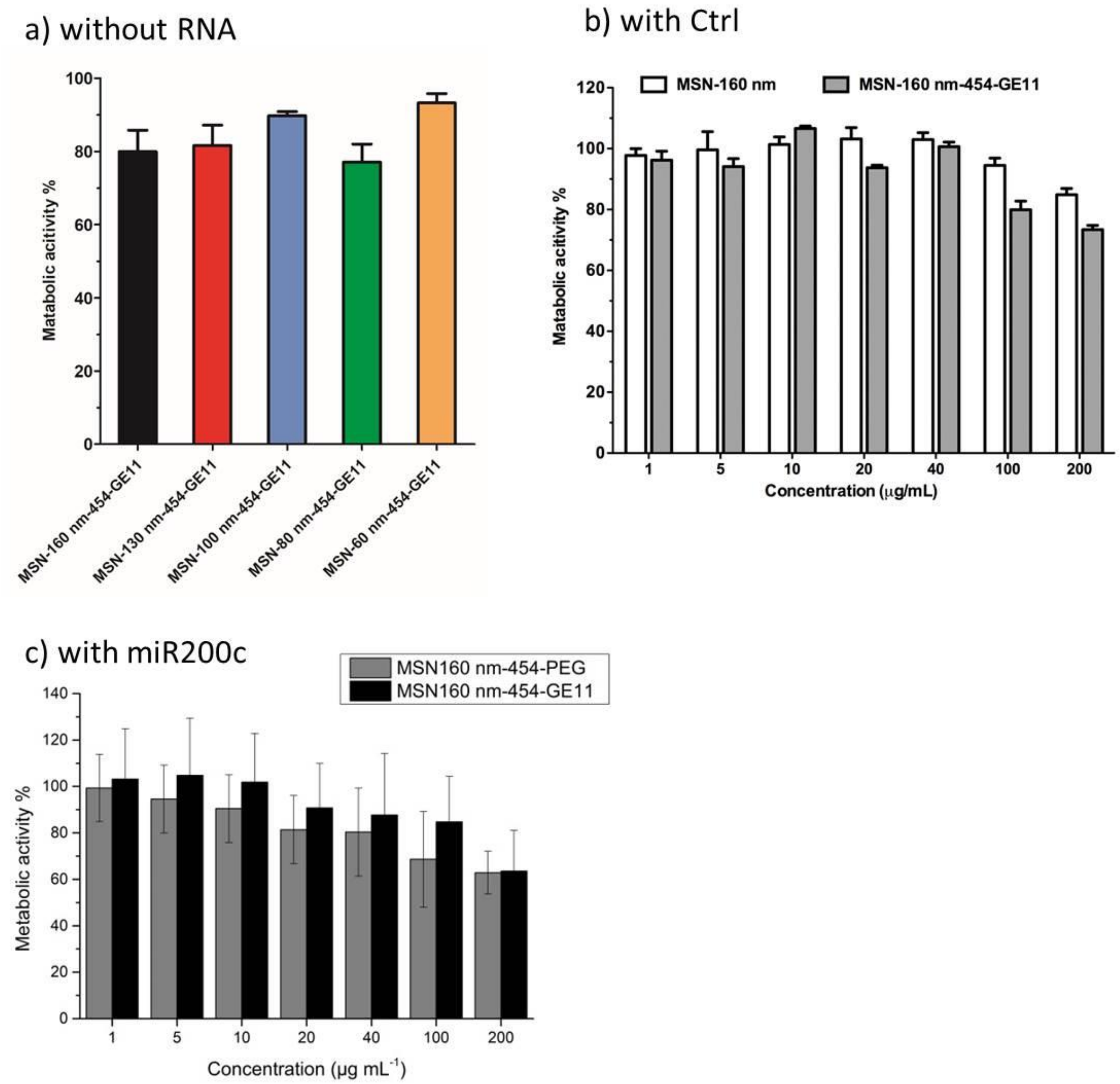

Figure S11. MTT cell viability study of MSN-454-GE11 samples on T24 cells after 48 h of transfection (cells were washed 45 min after incubation). a) MTT cell viability study of MSN-454-GE11 samples with various diameters using a concentration of 100 μg mL^{-1} as applied before in the in vitro cell experiments. b) MSN dose-dependent MTT assay with MSN160 nm (white) and MSN 160 nm-454-GE11 (grey) loaded with Ctrl. c) MTT assay of MSN-160 nm-454-GE11 in various concentrations in comparison with non-targeted sample MSN160 nm-454-PEG, both loaded with active miR200c (RNA concentration = 50 μg mg^{-1} of MSN carrier).

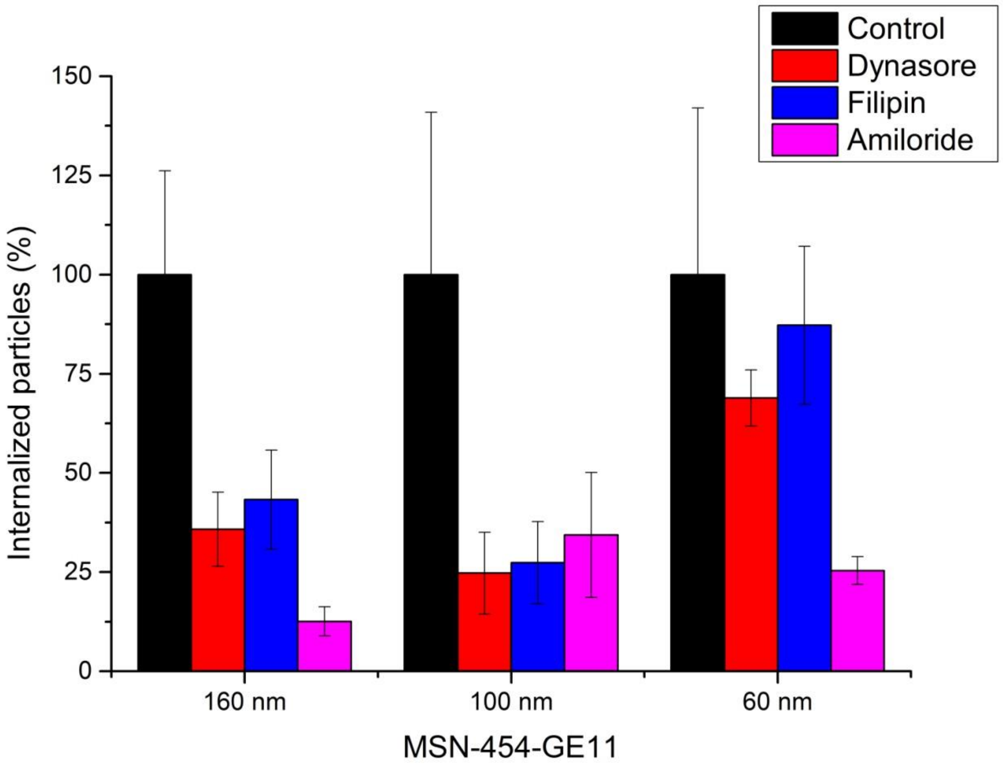

Figure S12. Inhibition results using Dynasore, 5-(*N*-Ethyl-N-isopropyl)amiloride and Filipin to inhibit different endocytosis pathways. We have performed confocal fluorescence microscopy experiments with subsequent image analysis for quantification of internalized particles and find that there are slight differences in uptake pathways. Control: uptake of particles in cells without inhibitor.

Flow cytometry of pure MSN samples

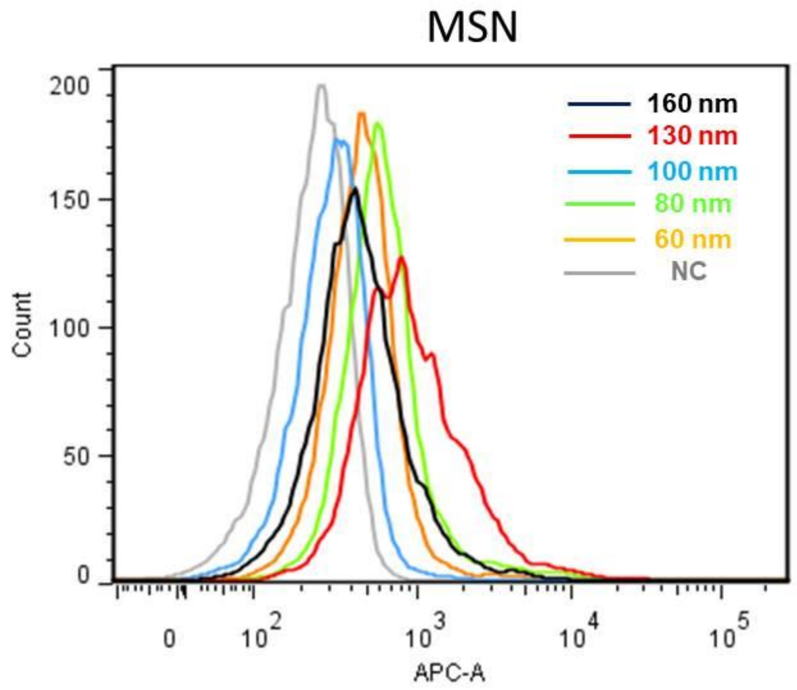

Figure S13: Cellular internalization of Atto-633 labeled pure MSN samples (without 454-GE11 polymer capping) with particle sizes in the range of 160 nm to 60 nm.
